# Epidemiological Characteristics of Peripheral T-Cell Lymphoma: A Population-Based Study

**DOI:** 10.3389/fonc.2022.863269

**Published:** 2022-07-13

**Authors:** Shuo Liu, Weiping Liu, Huichao Li, Lei Yang, Yuqin Song, Xi Zhang, Yangyang Cheng, Qingyu Li, Haoxin Li, Ning Wang, Jun Zhu, Jiafu Ji

**Affiliations:** ^1^ Key Laboratory of Carcinogenesis and Translational Research (Ministry of Education/Beijing), Beijing Office for Cancer Prevention and Control, Peking University Cancer Hospital and Institute, Beijing, China; ^2^ Key Laboratory of Carcinogenesis and Translational Research (Ministry of Education/Beijing), Department of Lymphoma, Peking University Cancer Hospital and Institute, Beijing, China; ^3^ Key Laboratory of Carcinogenesis and Translational Research (Ministry of Education/Beijing), Gastrointestinal Cancer Center, Peking University Cancer Hospital and Institute, Beijing, China

**Keywords:** non-Hodgkin lymphoma, peripheral T-cell lymphoma, epidemiology, cancer registry, incidence, mortality, survival

## Abstract

**Objects:**

The aim of this study is to explore the epidemiological characteristics of peripheral T-cell lymphoma in Beijing.

**Methods:**

All data were extracted from the Beijing Cancer Registry database from January 1, 2007, to December 31, 2018. Segi’s World Standard Population was used to estimate the age-standardized rate (ASR). Changes in trends were examined using joinpoint regression analysis. The observed survival was estimated by the Kaplan–Meier method. Relative survival was calculated using Ederer II and standardized using the Brenner method and International Cancer Survival Standard (ICSS) group 1 age structure. Stratified by gender, area, and histological type, incidence, mortality, and age of onset trends were observed in Beijing.

**Results:**

In Beijing, there were 801 new cases and 463 deaths of T-cell lymphoma from 2007 to 2018. Peripheral T-cell lymphoma not otherwise specified (PTCL-NOS) was the most prevalent subtype (37.45%), followed by angioimmunoblastic T-cell lymphoma (AITL; 20.35%), NK/T-cell lymphoma (NK/TCL; 17.60%), and anaplastic large cell lymphoma (ALCL; 10.24%). The crude incidence and mortality rates were 0.52 and 0.30 per 100,000 person-years, respectively, whereas the age-standardized incidence and mortality rates (ASIR and ASMR) were 0.35 and 0.18 per 100,000 person-years, respectively. Both ASIR and ASMR were more prevalent in men (0.48 and 0.24 per 100,000) and urban area (0.38 and 0.19 per 100,000) than in women (0.22 and 0.11 per 100,000) and rural area (0.30 and 0.15 per 100,000). The average annual percentage change (AAPC) of ASIR and ASMR was 5.72% (95% confidence interval (CI): 1.79%–9.81%) and 4.35% (95% CI: −0.09%–8.99%), respectively. The age-specific incidence rate increased with age and peaked at the age groups of 10–14 and 80–84. The mean and median age of onset increased between 2007 and 2018. In addition, it decreased after the age of onset was age standardization (*β = −0.41*, *P = 0.26*). The 5-year age-standardized relative survival was 39.02% for all patients, 58.14% for NK/TCL, 57.60% for ALCL, 31.38% for AITL, and 29.18% for PTCL-NOS.

**Conclusions:**

T-cell lymphoma incidence was rising, but survival was dismal in Beijing, indicating the need for improved early diagnosis and standardized treatment.

## Introduction

The age-standardized incidence and mortality of non-Hodgkin’s lymphoma (NHL) in China was 4.29 and 2.45 per 100,000, respectively, in 2016 (1). From 2006 to 2016, the incidence of NHL in China increased significantly (1). Moreover, according to the GLOBOCAN estimation, nearly 20% of the NHL worldwide occurred in China in 2020 (2). Peripheral T-cell lymphoma (PTCL) accounts for 24.38% of NHL (3).

PTCL, a group of heterogeneous mature T and NK cell diseases, is a rare malignancy with a poor prognosis (4). PTCL had geographical differences in epidemiological characteristics, for which the incidence and mortality were often higher in Asia than in Europe and North America. In Western countries, PTCL accounts for 5%–10% of all lymphomas. During 2011–2012, there were an estimated 8,380 new cases of PTCL in the United States, representing 6.1% of all new lymphoma cases, with the age-standardized incidence rate (ASIR) of 2.1 per 100,000 (5). In addition, between 2004 and 2014, 308 cases of PTCL accounted for 5.3% of all new patients with lymphoma in the UK population (6). In contrast, approximately 15% to 20% of lymphomas in Asia are categorized as PTCL. A total of 18.5% of 9426 cases of lymphoid neoplasms in Japan between 2007 and 2014 were PTCL, and 22.0% of 836 cases of all malignant lymphoma in Korea between 2005 and 2006 were PTCL (7, 8).

The Fourth Edition of the World Health Organization classification of lymphoid neoplasms described 27 subgroups, with PTCL not otherwise specified (PTCL-NOS), angioimmunoblastic T-cell lymphoma (AITL), anaplastic large cell lymphoma (ALCL), and natural killer/T-cell lymphoma (NK/TCL) being the most common subtypes (9). However, the prevalence of each subtype varies considerably between Western and Asian nations. In East Asia, extranodal NK/TCL was the most prevalent subtype, whereas in Europe and North America, PTCL-NOS was the most prevalent variety (7, 8, 10).

Because of the low prevalence and significant heterogeneous, published studies were predominantly multicenter and only represented a small percentage of the local population. There were few studies that examined the epidemiological characteristics of PTCL using population-based data. Therefore, we use the population-based cancer registry data in the present study, which aimed to explore the incidence, mortality, and age at diagnosis and analyze the survival outcome of PTCL in Beijing, China.

## Methods

### Data Source

All data were extracted from the Beijing Cancer Registry. Beijing Cancer Registry is a population-based cancer registry. It is responsible for collecting, analyzing, and publishing Beijing’s cancer surveillance data. There are 16 districts in Beijing. Districts of Dongchen, Xicheng, Chaoyang, Haidian, Fengtai, and Shijingshan were classified to urban areas, and rural areas include the district of Daxing, Shunyi, Fangshan, Changping, Mentougou, Tongzhou, Huairou, Pinggu, Miyun, and Yanqing. As defined by the International Agency for Research on Cancer/International Association of Cancer Registries (IARC/IACR), the incidence date corresponds to the date of the first consultation or admission to a hospital, clinic, or institution for the cancer in question. In addition, if admittance day was unavailable, then the date of the first cancer diagnosis by a physician or the date of the first pathology report was used (11). International rules for multiple primary cancers (ICD-O third edition) published by IARC/IACR that defined multiple primary cancers (12).

Incidence/mortality dates from January 1, 2007, to December 31, 2018, were included in this study. The range of codes extracted from the database was based on the International Classification of Diseases for Oncology third edition (ICD-O-3) morphology code by the 2016 WHO classification of mature lymphoid, histiocytic, and dendritic neoplasms (9). This study includes the following ICD-O-3 codes: 9700, 9701, 9702, 9705, 9708, 9709, 9714,9715, 9716, 9717, 9718, 9719, 9724, 9725, 9726, 9727, 9827,9831, 9834, and 9948. This research covers 155,244,248 person-years (77,900,807 for men and 77,343,441 for women, and 96,380,961 for urban areas and 58,863,287 for rural areas).

We utilize both passive and active follow-up methods to identify the vital status of cancer patients. The all-cause mortality database provided by Beijing Center for Diseases Prevention and Control (Beijing CDC) was used to link annually to the cancer registry incidence database by identifying information such as the patient’s ID number, name, sex, and date of birth. Community health cancer/station personnel followed up with the unlinked patients annually *via* telephone or family visits. In addition, patients who were lost to follow-up in the current year will have their key information matched with the CDC’s vital surveillance database in subsequent years, and death information will be collected if a patient’s death is reported in the CDC’s database in a given year.

Beijing Municipal Public Security Bureau and Beijing CDC supplied population and life table data stratified by age, gender, and area. From 2007 to 2018, the percentages of Beijing’s population in the 0–14, 15–44, 45–64, and 65 and older age groups were 10.43%, 42.85%, 31.98%, and 14.74%, respectively. In addition, the percentage of people aged 65 and above rose from 13.64% in 2007 to 17.81% in 2018. For detailed demographic structure, please refer to population pyramid for Beijing from 2007 to 2018 in **Supplementary Material 1**.

### Quality Control

Every hospital visit was reported to the Beijing Cancer Registry, and when different hospitals provided patients with conflicting diagnoses, their original reporting institutions had contacted to confirm and rectify the patient’s diagnosis. Medical records re-abstraction and annual training for cancer registrars in hospitals were implemented regularly to ensure the standardization of data coding and improve the data quality. IARC/IACR Cancer Registry Tools was utilized to validate the completeness, precision, and consistency of the variables (13). In cases identified as warning or error by the program, the reporting hospital would be contacted directly to verify the medical records. All peripheral T-cell lymphoma cases included in this study were morphology verified. None of them was a death certificate only case. The mortality-to-incidence ratio (M/I), which is one of the quality indicators of cancer registry data of this study, was 0.58. The percentage of individuals who were lost to follow-up in this study was 4.87%. In addition, the percentage of individuals who were lost to follow-up in time period of 2007–2010, 2011–2014, and 2015–2018 were 7.85%, 4.87%, and 2.17%, respectively.

### Statistical Analysis

T-test, Pearson’s Chi-squared test, and Wilcoxon rank-sum test were utilized to determine whether the general information differences between area and morphological type are statistically significant based on the distribution of the data. The burden of PTCL was reflected by the crude incidence rate, crude mortality rate, ASIR, and age-standardized mortality rate (ASMR) and stratified by sex, age group, and area (urban and rural areas). The ASRs were estimated by using Segi’s World Standard Population. T-cell lymphoma trend changes from 2007 to 2018 were analyzed using joinpoint regression. The average age of onset was indicated by mean ± SD, median, and standardized age of onset. A linear regression model was used to determine the trend of age at diagnosis. To determine whether the changes of age at diagnosis were statistically significant, the t-test was applied. The observed survival (OS) was estimated by the Kaplan–Meier method. Ederer II was used to evaluate relative survival (RS), and the ICSS group 1 age structure was standardized by the Brenner method. The last day of follow-up was December 31, 2020. Joinpoint (version 4.9.0.0) was used to examine the joinpoint model, whereas Stata (version 15.1) was utilized to analyze the other variables. The significance level used for interpreting the results was set at 0.05.

## Results

In Beijing, there were 6,379 new cases of NHL with identified histology subtype between 2007 and 2018, including 801 cases of PTCL, which accounted for 12.56%, and 3027 cases of NHL with identified histology subtype died, including 463 cases of PTCL, which accounted for 15.30%. The median age of PTCL was 60 years, and the male-to-female ratio was 2.3:1 (**Table 1**). In addition, the median age at diagnosis of AITL and PTCL-NOS was 64 and 62 years, respectively. They were followed by NK/TCL, which was 55 years old. The median age at diagnosis of ALCL was 49 years. The most prevalent subtype was PTCL-NOS (37.45%), followed by AITL (20.35%), NK/TCL (17.60%), and ALCL (10.24%) (**Table 1**). Only the median age of diagnosis has shown a statistically significant difference between urban and rural areas among all general parameters. In rural areas, the median age of diagnosis was younger than in urban areas (58 vs. 60 years, Z = 2.86, P = 0.004).

**Table 1:** Baseline characteristics of 801 patients with peripheral T-cell lymphoma.

	All areas	Urban area	Rural area	Statistic test
	(n = 801, %)	(n = 560, %)	(n = 241, %)	
Sex				
Male	557, 69.54	391, 69.82	166, 68.88	χ^2^ = 0.07
Female	244, 30.46	169, 30.18	75, 31.12	P = 0.791
Age				
Median* (years)	60	60	58	Z = 2.86
Range (years)	1–102	1–102	1–85	P=0.004
Mean	57.15 ± 18.81	58.45 ± 18.61	54.13 ± 18.97	
Ethnicity				
Han	741, 92.51	516, 92.14	225, 93.36	χ^2^ = 0.36
Others	60, 7.49	44, 7.86	16, 6.64	P = 0.548
Histological type				
PTCL-NOS	300, 37.45	203, 36.25	97, 40.25	χ^2^ = 3.90
AITL	163, 20.35	124, 22.14	39, 16.18	P = 0.420
NK/TCL	141, 17.60	96, 17.14	45, 18.67	
ALCL	82, 10.24	57, 10.18	25, 10.37	
Others	115, 14.36	80, 14.29	35, 14.53	
Diagnosis				
2007–2010	191, 23.85	142, 25.36	49, 20.34	χ^2^ = 3.35
2011–2014	287, 35.83	191, 34.11	96, 39.83	P = 0.188
2015–2018	323, 40.32	227, 40.53	96, 39.83	
Treatment hospital				
Tertiary	757, 94.51	533, 95.18	224, 92.95	χ^2^ = 1.62
Non-tertiary	44, 5.49	27, 4.82	17, 7.05	P = 0.203

AITL, angioimmunoblastic T-cell lymphoma; ALCL, anaplastic large cell lymphoma; NK/TCL, Natural killer/T-cell lymphoma; PTCL-NOS, peripheral T-cell lymphoma not otherwise specified *Statistically significant.

### Incidence and Mortality

The crude incidence and mortality rates per 100,000 person-years were 0.52 and 0.30, respectively, whereas the ASIR and ASMR were 0.35 and 0.18 per 100,000 person-years, respectively (**Table 2**). The ASIR was higher among men (0.48 per 100,000) and urban areas (0.38 per 100,000) than among women (0.22 per 100,000) and rural areas (0.30 per 100,000), respectively. The ASMR of men (0.24 per 100,000) and urban areas (0.19 per 100,000) were higher than those of women (0.11 per 100,000) and rural areas (0.15 per 100,000), respectively.

**Table 2:** Incidence and mortality of peripheral T-cell lymphoma in Beijing from 2007 to 2018.

	Areas	Sex	Cases	Crude Rate(1/100,000)	ASR(1/100,000)
Incidence	All Areas	Male	557	0.72	0.48
		Female	244	0.32	0.22
		Both	801	0.52	0.35
	Urban	Male	391	0.81	0.52
		Female	169	0.35	0.25
		Both	560	0.58	0.38
	Rural	Male	166	0.56	0.43
		Female	75	0.26	0.18
		Both	241	0.41	0.30
Mortality	All Areas	Male	315	0.40	0.24
		Female	148	0.19	0.11
		Both	463	0.30	0.18
	Urban	Male	224	0.46	0.28
		Female	108	0.23	0.15
		Both	332	0.34	0.19
	Rural	Male	91	0.31	0.22
		Female	40	0.14	0.10
		Both	131	0.22	0.15

ASR, age-standardized rate.

The age-specific incidence rate of T-cell lymphoma showed the first peak in the 10- to 14-year age group, increased steeply from that age group, and reached a second peak in the 80- to 84-year age group (**Table 3**). The highest age-specific incidence rate for men and urban was at 80- to 84-year age group, whereas the highest rate for women and rural area were at 75- to 79-year age group. Except for the age groups of 5–9 years and 10–14 years, men had a greater age-specific incidence rate than women. After the age group of 35-39 years, urban areas had a higher age-specific incidence rate than rural areas. The age-specific T-cell lymphoma mortality rate is relatively low before 35 years old and has steadily climbed since then, reaching its peak between the age group of 80–84 years.

**Table 3:** Age-specific incidence and mortality rates by sex and area of peripheral T-cell lymphoma in Beijing from 2007 to 2018 (1/100,000).

Age group	Incidence					Mortality				
	Total	Male	Female	Urban	Rural	Total	Male	Female	Urban	Rural
0–4	0.11	0.12	0.09	0.05	0.19	0.03	0.03	0.03	0.03	0.04
5–9	0.16	0.15	0.16	0.20	0.10	0.04	0.04	0.04	0.00	0.10
10–14	0.22	0.21	0.23	0.28	0.15	0.00	0.00	0.00	0.00	0.00
15–19	0.18	0.33	0.03	0.24	0.11	0.08	0.09	0.06	0.11	0.04
20–24	0.18	0.24	0.11	0.16	0.21	0.09	0.12	0.05	0.06	0.14
25–29	0.19	0.24	0.14	0.19	0.20	0.08	0.07	0.08	0.09	0.06
30–34	0.17	0.29	0.05	0.16	0.20	0.08	0.13	0.03	0.08	0.09
35–39	0.31	0.43	0.18	0.36	0.23	0.14	0.17	0.11	0.19	0.07
40–44	0.33	0.43	0.22	0.36	0.28	0.13	0.18	0.09	0.11	0.16
45–49	0.42	0.67	0.16	0.52	0.28	0.20	0.32	0.07	0.22	0.17
50–54	0.53	0.78	0.28	0.61	0.40	0.20	0.33	0.07	0.23	0.15
55–59	0.69	0.98	0.42	0.76	0.59	0.40	0.62	0.18	0.44	0.33
60–64	0.99	1.45	0.54	1.06	0.87	0.63	0.91	0.36	0.76	0.41
65–69	1.16	1.54	0.82	1.26	1.01	0.72	0.95	0.51	0.85	0.51
70–74	1.34	1.72	1.00	1.36	1.30	0.90	1.08	0.73	0.83	1.04
75–79	1.67	2.23	1.17	1.74	1.52	1.14	1.37	0.94	1.13	1.18
80–84	1.69	2.68	0.77	2.06	0.68	1.63	2.42	0.89	2.02	0.57
85–89	0.84	1.33	0.41	0.97	0.38	0.79	1.23	0.41	0.97	0.19

**Figure 1:**
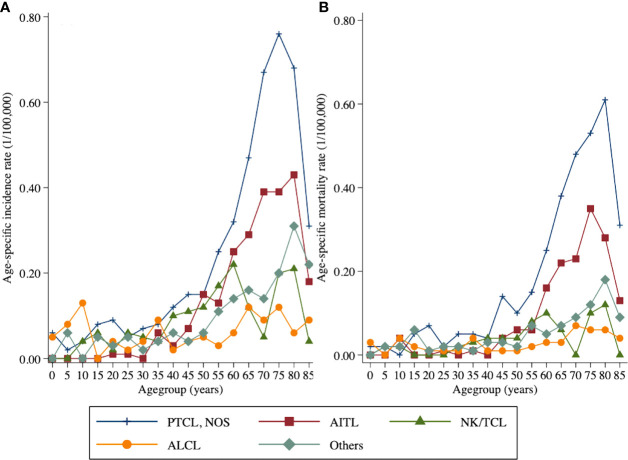
Age-specific incidence and mortality rates by histology subtype of peripheral T-cell lymphoma in Beijing from 2007 to 2018: **(A)** incidence and **(B)** mortality.

The age-specific incidence rates of T-cell lymphoma varied significantly among histological subgroups. The age-specific incidence rate of PTCL-NOS and AITL generally increased with age and peaked at 75- to 79-year and 80- to 84-year age group, respectively. In addition, the highest age-specific incidence rate of NK/TCL was observed in those aged 60–64 years. For ALCL, the age-specific incidence rate first peaked at 10- to 14-year age group then peaked at 35- to 39-year age group and 75- to 79-year age group, respectively. As for the age-specific mortality rate of four main histology subtypes, which was increased with age and peaked at 80- to 84-year age group for PTCL- NOS and NK/TCL, 75- to 79-year age group for AITL, and 70- to 74-year age group for ALCL (**Figure 1**; **Supplementary Material 2**).

### Survival Outcome

**Figure 2:**
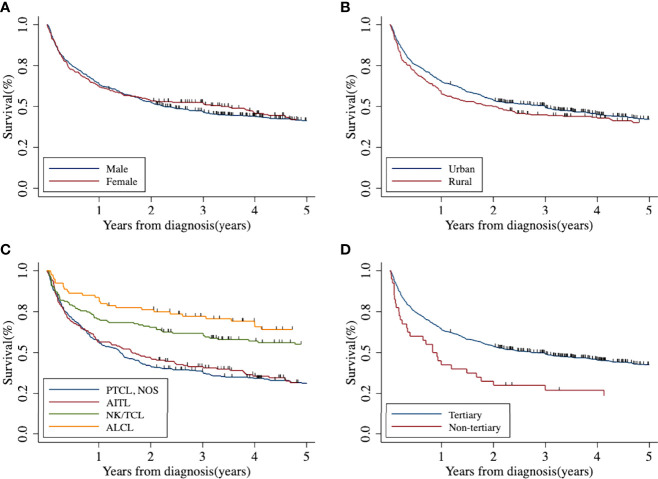
Five-year Kaplan–Meier survival curve of peripheral T-cell lymphoma in Beijing from 2007 to 2018: **(A)** by sex; **(B)** by area; **(C)** by histological type; **(D)** by treatment hospital. AITL, angioimmunoblastic T-cell lymphoma; ALCL, anaplastic large cell lymphoma; NK/TCL, natural killer/T-cell lymphoma; PTCL-NOS, peripheral T-cell lymphoma not otherwise specified.

The 5-year OS and RS of T-cell lymphoma in Beijing were 41.42% (95% CI: 37.85%–44.95%) and 44.37% (95% CI: 40.55%–48.16%), respectively. After age standardization, the RS was 39.02% (95% CI: 34.50%–43.57%). The 5-year Kaplan–Meier survival curve of peripheral T-cell lymphoma in Beijing stratified by sex, area, histology type, and treatment hospital was demonstrated in **Figure 2**. The age-standardized RS of men (39.55%; 95% CI: 33.99%–45.15%) and urban areas (40.00%; 95% CI: 34.67%–45.35%) was slightly higher than that of women (38.97%; 95% CI: 31.06%–46.90%) and rural areas (37.24%; 95% CI: 27.94%–46.72%) (**Table 4**).

**Table 4:** Five-year survival by sex, area, histological type, and treatment hospital of peripheral T-cell lymphoma in Beijing from 2007 to 2018.

	OS(%, 95% CI)	RS(%, 95% CI)	Age-standardized RS(%, 95% CI)
Sex			
Male	41.02 (36.77–45.22)	44.27 (39.69–48.80)	39.55 (33.99–45.15)
Female	42.32 (35.70–48.78)	44.59 (37.61–51.40)	38.97 (31.06–46.90)
Area			
Urban	41.96 (37.65–46.21)	45.31 (40.65–49.89)	40.00 (34.67–45.35)
Rural	40.10 (33.71–46.40)	42.16 (35.45–48.78)	37.24 (27.94–46.72)
Histological type			
PTCL, NOS	30.99 (25.69–36.43)	33.28 (27.59–39.13)	29.18 (23.03–35.67)
AITL	31.80 (24.26–39.58)	34.03 (25.96–42.37)	31.38 (23.10–40.12)
NKTCL	54.93 (46.06–62.95)	58.00 (48.63–66.47)	58.14 (42.63–71.80)
ALCL	63.86 (51.61–73.78)	67.15 (54.27–77.59)	57.60 (36.88–75.26)
Treatment hospital			
Tertiary	42.37 (38.69–46.00)	45.36 (41.41–49.24)	40.01 (35.25–44.79)
Non–tertiary	23.70 (11.67–38.10)	25.87 (12.74–41.60)	25.39 (11.66–42.15)
Time period			
2007–2010	45.72 (38.36–52.77)	49.31 (41.37–56.91)	45.95 (36.43–55.24)
2011–2014	38.55 (32.82–44.23)	41.18 (35.06–47.25)	36.53 (29.52–43.66)
2015–2018	41.27 (35.28–47.15)	44.15 (37.74–50.44)	36.72 (28.90–44.71)

OS, observed survival; RS, relative survival; AITL, angioimmunoblastic T-cell lymphoma; ALCL, anaplastic large cell lymphoma; NK/TCL, natural killer/T-cell lymphoma; PTCL-NOS, peripheral T-cell lymphoma not otherwise specified.

NK/TCL and ALCL had better survival, with 5-year age-standardized RS of 58.14% (95% CI: 42.63%–71.80%) and 57.60% (95% CI: 36.88%–75.26%), respectively. The survival of PTCL-NOS and AITL was relatively low, at 29.18% (95% CI: 23.03%–35.67%) and 31.38% (95% CI: 23.10%–40.12%), respectively.

The patients who received treatment in tertiary hospitals had better survival than the non-tertiary hospital with the 5-year age-standardized RS of 40.01% (35.25%–44.79%) and 25.39% (11.66%–42.15%), respectively.

### Trends of the Age of Onset and Age-Specific Incidence Rate

**Figure 3:**
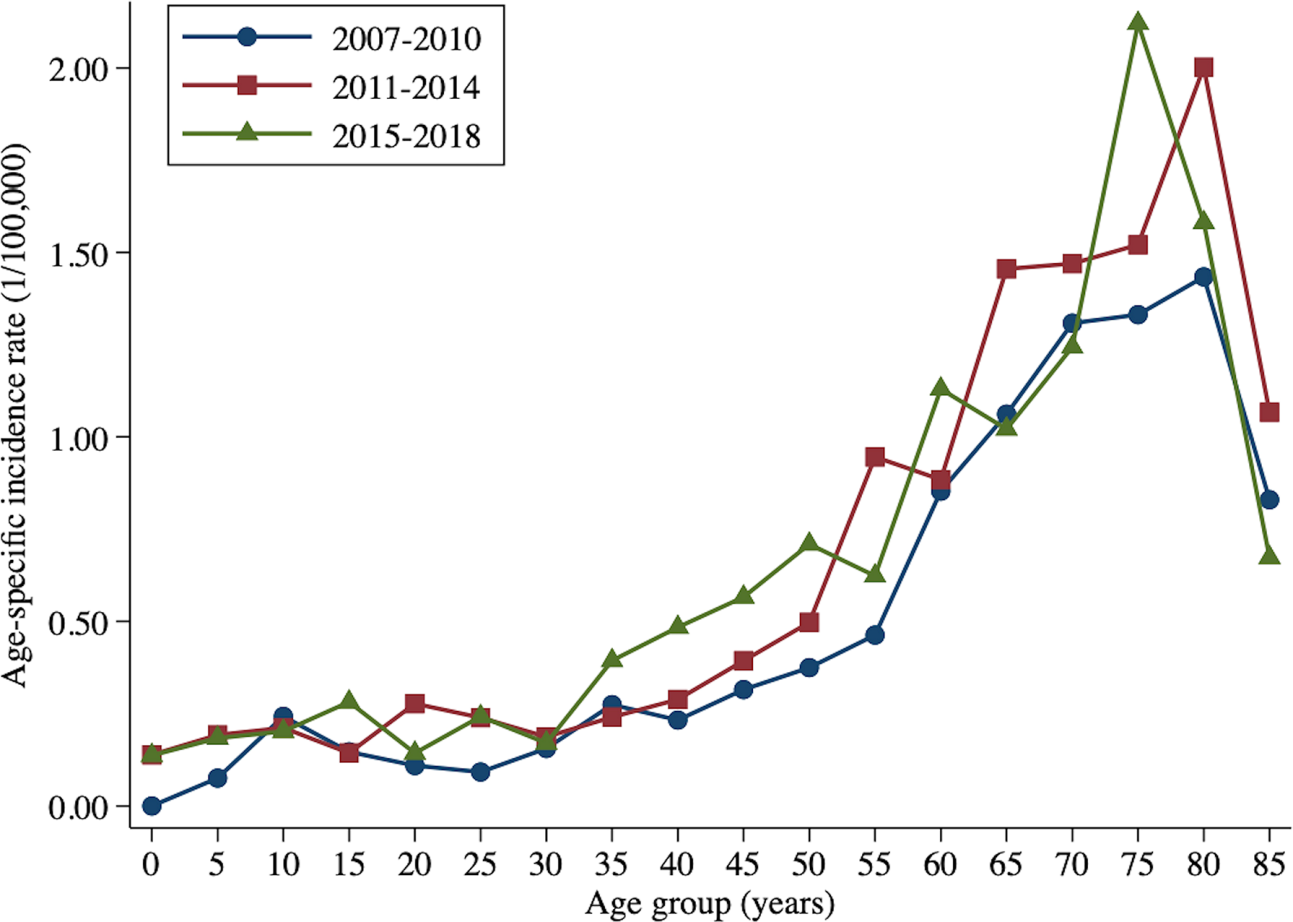
Age-specific incidence rate of peripheral T-cell lymphoma in Beijing.

**Figure 4:**
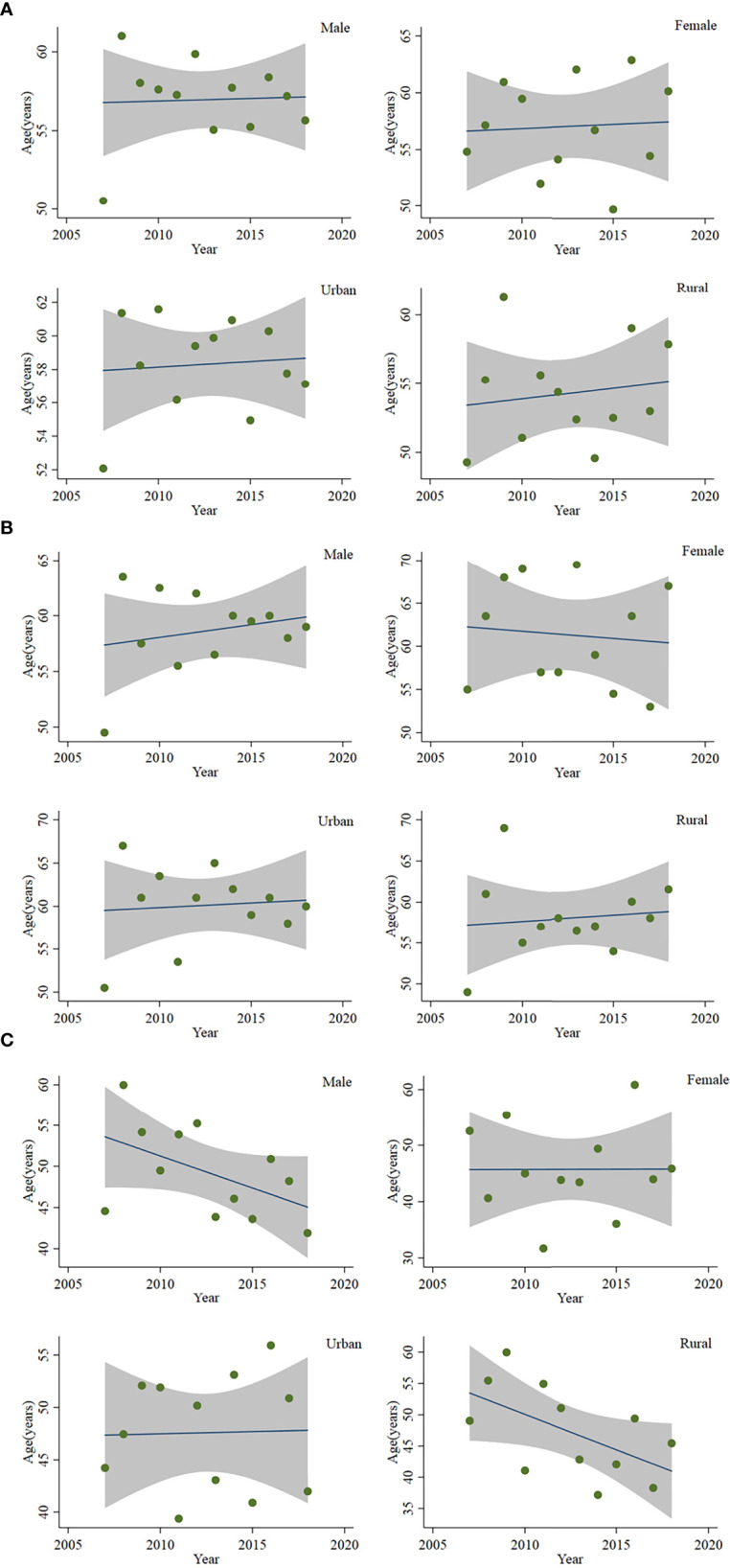
Age of onset of patients with peripheral T-cell lymphoma in Beijing from 2007 to 2018: **(A)** mean age of onset, **(B)** median age of onset, and **(C)** standardized mean age of onset.

From 2007 to 2010, the age-specific incidence rate of peripheral T-cell lymphoma in Beijing peaked at the 80- to 84-year age group. From 2015 to 2017, however, the age-specific incidence rate moved forward to the 74- to 79-year age group (**Figure 3**). The mean and median age of onset for patients with T-cell lymphoma in Beijing increased from 51.63 and 50 years old to 57.33 and 60 years old, respectively (**Table 5**). Nevertheless, the standardized mean age of onset showed a decreasing pattern from 2007 to 2018 in Beijing. The result is not statistically significant, though. For men and urban areas, the age of onset trends exhibited the same features as the population overall (**Figure 4**). Specifically in rural regions, the usual age of onset declined by 1.14 years annually. Nevertheless, the outcome is only boundary statistically significant.

**Table 5:** Trends of age of onset of peripheral T-cell lymphoma in Beijing from 2007 to 2018 (years).

year	Total			Male			Female			Urban			Rural		
	Mean	Median	Standardized	Mean	Median	Standardized	Mean	Median	Standardized	Mean	Median	Standardized	Mean	Median	Standardized
2007	51.63	50.00	46.14	50.53	49.50	44.56	54.82	55.00	52.57	52.08	50.50	44.24	49.29	49.00	49.04
2008	59.50	63.50	51.45	61.00	63.50	59.97	57.14	63.50	40.62	61.36	67.00	47.45	55.27	61.00	55.46
2009	58.97	62.00	54.58	58.03	57.50	54.16	60.95	68.00	55.40	58.24	61.00	52.09	61.29	69.00	59.97
2010	58.21	63.00	47.82	57.61	62.50	49.49	59.47	69.00	45.00	61.58	63.50	51.91	51.06	55.00	41.09
2011	55.95	56.00	46.22	57.27	55.50	53.88	52.00	57.00	31.71	56.17	53.50	39.38	55.63	57.00	54.94
2012	57.87	59.00	50.89	59.86	62.00	55.23	54.12	57.00	43.82	59.40	61.00	50.18	54.39	58.00	51.05
2013	57.21	61.00	43.60	55.05	56.50	43.85	62.04	69.50	43.42	59.89	65.00	43.06	52.40	56.50	42.83
2014	57.45	59.50	46.56	57.72	60.00	46.07	56.70	59.00	49.38	60.93	62.00	53.12	49.59	57.00	37.18
2015	54.00	58.50	41.43	55.24	59.50	43.60	49.72	54.50	36.03	54.94	59.00	40.90	52.52	54.00	42.06
2016	59.96	61.00	53.92	58.38	60.00	50.89	62.87	63.50	60.86	60.28	61.00	55.92	59.00	60.00	49.38
2017	56.49	58.00	46.88	57.20	58.00	48.21	54.42	53.00	43.96	57.76	58.00	50.88	53.00	58.00	38.29
2018	57.33	60.00	43.09	55.65	59.00	41.90	60.13	67.00	45.84	57.12	60.00	41.98	57.83	61.50	45.43
Total	57.15	60.00	47.08	56.88	59.00	48.15	57.77	61.00	45.27	58.45	60.00	47.39	54.13	58.00	45.62
*β*	0.09	0.15	-0.41	0.03	0.23	-0.78	0.07	-0.16	0.01	0.07	0.11	0.04	0.16	0.14	-1.14
*t*	0.44	0.48	-1.19	0.14	0.72	-1.82	0.2	-0.31	0.01	0.26	0.27	0.09	0.49	0.34	-2.16
*P*	0.67	0.64	0.26	0.89	0.49	0.10	0.85	0.76	0.99	0.80	0.80	0.93	0.64	0.74	0.06

### Trends of Incidence and Mortality Rates

Both incidence and mortality of T-cell lymphoma increased in Beijing between 2007 and 2018 (**Table 6**). The average annual percentage change (AAPC) of ASIR and ASMR of T-cell lymphoma in Beijing from 2007 to 2018 was 5.72% (1.79%–9.81%) and 4.35% (−0.09%–8.99%), respectively. The AAPC of ASIR for men (6.26%; 95% CI: 1.44%–11.31%) was higher than that of women (4.21%; 95% CI: −0.28%–8.91%), and the AAPC of ASMR for women (4.77%; 95% CI: −6.10%–16.92%) was slightly higher than that of men (4.71%; 95% CI: 0.59%–9.00%). The AAPC of ASIR and ASMR for rural areas (7.10%, 95% CI: −2.43%–17.56%; and 8.07%, 95% CI 0.07–16.70%) was higher than that of urban areas (4.42%, 95% CI: 0.18%–8.83%; and 2.75%, 95% CI: −2.47%–8.25%).

**Table 6:** Age-standardized incidence and mortality rates of patients with peripheral T-cell lymphoma in Beijing.

	Area	Sex	2007–2010 (1/100,000)	2011–2014 (1/100,000)	2015–2018 (1/100,000)
Incidence	Total	Male	0.37	0.53	0.56
		Female	0.18	0.23	0.24
		Both	0.27	0.38	0.40
	Urban	Male	0.43	0.54	0.59
		Female	0.22	0.23	0.28
		Both	0.32	0.38	0.43
	Rural	Male	0.27	0.51	0.52
		Female	0.12	0.23	0.17
		Both	0.20	0.37	0.35
Mortality	Total	Male	0.21	0.25	0.32
		Female	0.09	0.14	0.14
		Both	0.15	0.20	0.23
	Urban	Male	0.24	0.24	0.37
		Female	0.11	0.16	0.16
		Both	0.17	0.20	0.26
	Rural	Male	0.14	0.28	0.24
		Female	0.04	0.12	0.12
		Both	0.09	0.20	0.18

**Table 7** shows the incidence and mortality of peripheral T-cell lymphoma by histological subtypes in Beijing from 2007 to 2018. Among the four major histology types, NK/TCL grew the rapidly in ASIR and ASMR. The AAPC of ASIR and ASMR were 25.08% (95% CI: 4.77%–49.32%) and 30.15% (95% CI: 17.52%–44.15%). Followed by AITL, the AAPC of ASIR and ASMR were 23.06% (95% CI: −32.90%–125.68%) and 24.91% (95% CI: −29.66%–121.80%). However, the AAPC is not statistically significant. The ASIR and ASMR of PTCL, NOS, and ALCL are relatively stable during the past decade. The AAPC were 0.38% (95% CI: −6.59%–7.87) and −0.40% (95% CI: −7.83%–7.63%) for incidence and −1.31% (95% CI: −7.18%–4.92%) and 4.37% (95% CI: −6.01%–15.11%) for mortality, respectively.

**Table 7:** Incidence and mortality by histology type of patients with peripheral T-cell lymphoma in Beijing from 2007 to 2018.

Year	PTCL-NOS			AITL			NK/TCL			ALCL			Others		
	Cases	Rate (1/100,000)	ASR (1/100,000)	Cases	Rate (1/100,000)	ASR (1/100,000)	Cases	Rate (1/100,000)	ASR (1/100,000)	Cases	Rate (1/100,000)	ASR (1/100,000)	Cases	Rate (1/100,000)	ASR (1/100,000)
Incidence															
2007–2010	87	0.177	0.112	29	0.059	0.038	13	0.026	0.016	24	0.049	0.049	38	0.077	0.056
2011–2014	125	0.241	0.154	58	0.112	0.061	45	0.087	0.063	29	0.056	0.067	30	0.058	0.032
2015–2018	88	0.162	0.115	76	0.140	0.072	83	0.153	0.107	29	0.053	0.042	47	0.087	0.064
Mortality															
2007–2010	56	0.114	0.074	18	0.004	0.024	4	0.001	0.004	8	0.002	0.011	21	0.004	0.026
2011–2014	88	0.170	0.094	29	0.006	0.030	16	0.003	0.018	13	0.003	0.021	20	0.004	0.024
2015–2018	70	0.129	0.066	51	0.009	0.042	37	0.007	0.042	13	0.002	0.017	19	0.004	0.024

AITL, angioimmunoblastic T-cell lymphoma; ALCL, anaplastic large cell lymphoma; NKTCL, natural killer/T-cell lymphoma; PTCL-NOS, peripheral T-cell lymphoma not otherwise specified; ASR, age-standardized rate.

**Figure 5:**
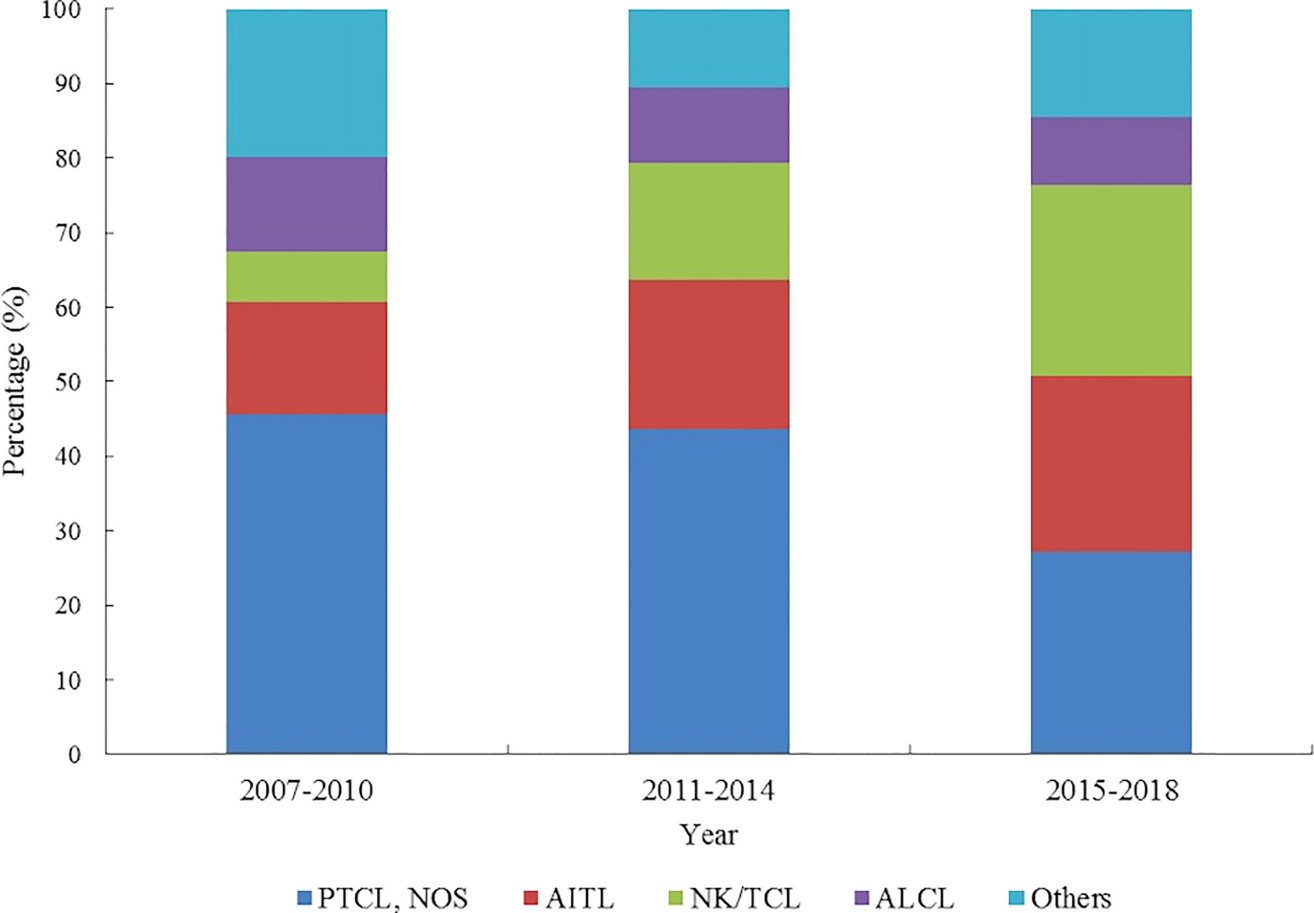
Histology type proportion changes of patients with peripheral T-cell lymphoma in Beijing from 2007 to 2018.

The proportion of NK/TCL and AITL increased from 6.81% and 15.18% to 25.70% and 23.53%, respectively. However, the proportion of PTCL-NOS, and ALCL decreased from 45.55% and 12.56% to 27.24% and 8.98%, respectively (**Figure 5**; **Supplementary Material 3**).

## Discussion

The present study provided a comprehensive evaluation of age- and sex-specific incidence, mortality, and survival outcomes of PTCL using population-based data. PTCL-NOS, AITL, NK/TCL, and ALCL constituted the majority of PTCLs. Incidence and mortality rates of certain subtypes varied substantially between age groups. The incidence and mortality rates were greater among men and urban residents than among women and rural residents. Despite considerable urban–rural disparities in the incidence and mortality of PTCL, the survival outcome was inferior in both areas.

The crude and ASIR of PTCL for Beijing from 2007 to 2018 was 0.52 and 0.35 per 100,000 person-years. Compared with other development countries, it was comparatively low. Lee et al. reported the T- and NK-cell neoplasms crude incidence rate and ASIR was 0.97 and 0.82 per 100,000 person year based on the Korea Central Cancer Registry annual report data between 1999 and 2012 (14). In addition, the result from UK’s Haematological Malignancy Research Network demonstrated that the crude incidence rate and ASIR by European population structure of T-cell lymphoma was 1.08 and 0.92 per 100,000 person year from 2004 to 2014 (6). In addition, the ASIR by the US standard population of T/NK-cell lymphoid neoplasm was 2.1 per 100,000 from 2011 to 2012, according to the Surveillance, Epidemiology, and End Results (SEER) database (5). These differences may be partially attributable to the capacity for diagnosis and pathology. Over 40% of lymphoma cases in China were classified as non-specific NHL (1). To improve the early diagnosis and treatment of PTCL and other subtypes of lymphoma in China, it is recommended to strengthen the country’s pathological and clinical diagnostic and treatment capacities.

The lymphoma burden is significantly influenced by socioeconomic disparities. A study revealed that the incidence of lymphoma in urban areas of the United States increased approximately 1.5-fold compared with rural areas (15). Similarly, the ASIR of non-Hodgkin lymphoma in men and women was 5.5 and 3.9 per 100,000 population in urban areas and 4.3 and 2.9 per 100,000 population in rural areas in China, respectively (16). Notably, the survival outcome of patients residing in urban areas was better than that of patients residing in rural areas, which may be partially explained by their greater access to healthcare (17). In the present study, the incidence and mortality of PTCL were higher in urban areas than in rural areas, whereas the survival outcome of patients with PTCL was better in urban areas. These findings supported the establishment of a precise regional strategy of disease control and prevention.

Age and sex were associated with diverse epidemiological characteristics of lymphoma. A study involving 13,107 patients with PTCL in the United States showed a male predominance across all ethnic groups. Moreover, patients with PTCL-NOS and AITL were older than those with ALCL and NK/TCL (median age: 65 years vs. 69 years and vs. 56 years vs. 54 years) (18). Similarly, the median age was 35.6 years for anaplastic lymphomakinase (ALK)–positive ALCL and 71.9 years for AITL in the United Kingdom (19). In addition, men had a higher ASIR of PTCL than women (1.14 vs. 0.74, per 100,000 population) (6). Although the median age was 60 years in the present study, older individuals were more likely to develop AITL, whereas younger individuals were more likely to develop ALK-positive ALCL. Consequently, additional research is required to identify the critical factors influencing PTCL prevalence among different age and sex groups.

Except for ALK-positive ALCL, the prognosis for almost all PTCL subtypes is dismal. A report involving 1,314 patients with PTCL and NK/TCL showed that the 5-year OS was 70% for ALK-positive ALCL and 32% for PTCL-NOS, angioimmunoblastic, and NK/TCL (10). Moreover, patients with refractory and relapsed disease had an inferior prognosis with a median OS of 5.8 months (19, 20). A study included 13,107 patients with PTCL and demonstrated that the median survival time ranged from 22 to 49 months and varied by race (18). Despite the use of new drugs and hematopoietic stem cell transplantation, the prognosis for PTCL has not improved significantly (21–26). The 5-year RS changed from 29.6% in 1993–1997 to 33.2% in 2003–2006 in Japan (27), whereas the 5-year OS changed from 41% in 1996–2000 to 51% in 2011–2015 in China (28), but neither difference was statistically significant. In the current study, chemotherapy-sensitive subtypes such as ALCL had a favorable prognosis, whereas chemotherapy-resistant subtypes such as PTCL-NOS had inferior survival outcomes. In addition, except for the overestimation of PTCL survival in 2007 to 2010, the age-standardized RS of PTCL did not improve from the time period of 2015–2018 and 2011–2014. These findings highlight the need to develop effective treatment strategies, especially for chemotherapy-resistant subtypes. Moreover, patients with PTCL treated in tertiary hospitals had a significantly higher survival rate than those treated in non-tertiary hospitals. It could be due to the complexity of the biologically and clinically diverse group of such rare tumors. In addition, it was suggested that non-tertiary medical facilities should provide regular training for specialists to promote standardized diagnosis and treatment.

According to the analysis of the global burden of disease 2016, the incidence of non-Hodgkin lymphoma increased significantly from 2006 to 2016 (ASIR per 100,000 population, 2.74 in 2006 vs. 4.29 in 2016), whereas the mortality remained stable (ASMR per 100,000 population, 2.21 in 2006 vs. 2.45 in 2016) (1). In both urban and rural areas, lymphoma and myeloma mortality increased significantly (29). Consistent with previous studies, rural areas had 1.6 times the AAPC of ASIR and 2.9 times the AAPC of ASMR than urban areas in the present study. These findings confirmed the upward PTCL burden and supported improved access to health services, particularly in rural areas.

The age-specific incidence rate of PTCL peaked at 80–84 years during the 2007–2010 and 2011–2014 time periods, but peaked at 74–79 years during 2015–2017. Moreover, after mitigating the effect of population aging, the age at diagnosis of PTCL showed a decreasing pattern, particularly in men and rural populations, although the result is not statistically significant. After adjusting for age structure, the mean age of patients diagnosed with cancer decreased by 0.13 years per year in China, particularly among women, indicating a younger trend for cancer incidence in China (30). The altering histology composition may partially explain the decreasing age of PTCL onset. Among the four primary histology types, the proportion and incidence of NK/TCL have grown the quickest. Moreover, in addition to this, the youth-oriented trend of PTCL may be due to etiology factors or the improvement of diagnosis, both of which require additional observation and verification.

There were several limitations in the present study. First, not every case in our study had its histology type confirmed by a reference institution. It would only review and confirm a patient’s diagnosis if different hospitals provided contradictory diagnoses for the same patient.

Second, because the extremely small number of PTCL cases among ethnicities other than Han, it was impossible to compare epidemiological characteristics across ethnic groups. Third, the time trend of rare subtypes, such as enteropathy-associated T-cell lymphoma, was not evaluated. Fourth, the impact of the treatment model on survival could not be determined due to the absence of treatment information in the registry database. Fifth, despite the fact that this study used a mix of active and passive follow-up, it is inevitable that some patients’ survival data cannot be obtained. As a result of that, the survival would be overestimated. Especially for the patients diagnosed in the early year.

In conclusion, the incidence and mortality of PTCL in Beijing exhibited an upward trend. Notable was the relatively low survival rate. More attention and further studies are needed in etiological studies, early detection and diagnosis, standardized treatment, and other aspects of this area.

## Data Availability Statement

The original contributions presented in the study are included in the article/**Supplementary Material**. Further inquiries can be directed to the corresponding authors.

## Author Contributions

SL and WL conceived and designed the study, analyzed the data, and drafted and revised the paper. XZ, YC, QL, and HL (9th Author) prepared and analyzed the data. HL (3rd Author), LY, and YS drafted and revised the paper. NW, JZ, and JJ designed the study, interpreted the results, and drafted and revised the paper. All authors provided critical comments on the manuscript. All authors read and approved the final manuscript.

## Funding

This study was funded by Beijing Hospitals Authority Youth Programme (No. QML20211102). The funder did not participate in any part of the study from study design to approval of the manuscript, except for supporting this project.

## Conflict of Interest

The authors declare that the research was conducted in the absence of any commercial or financial relationships that could be construed as a potential conflict of interest.

## Publisher’s Note

All claims expressed in this article are solely those of the authors and do not necessarily represent those of their affiliated organizations, or those of the publisher, the editors and the reviewers. Any product that may be evaluated in this article, or claim that may be made by its manufacturer, is not guaranteed or endorsed by the publisher.
