# Supplementary material for: Epidemiological Characteristics of Peripheral T-Cell Lymphoma: A Population-Based Study
Source: Front Oncol. 2022 Jul 13;12:863269. doi: 10.3389/fonc.2022.863269 (PMC9326059; doi:10.3389/fonc.2022.863269)
Supplement: Supplementary file 1 [file DataSheet_1.docx]

Supplementary Material 1

Population pyramid of Beijing from 2007 to 2018

**
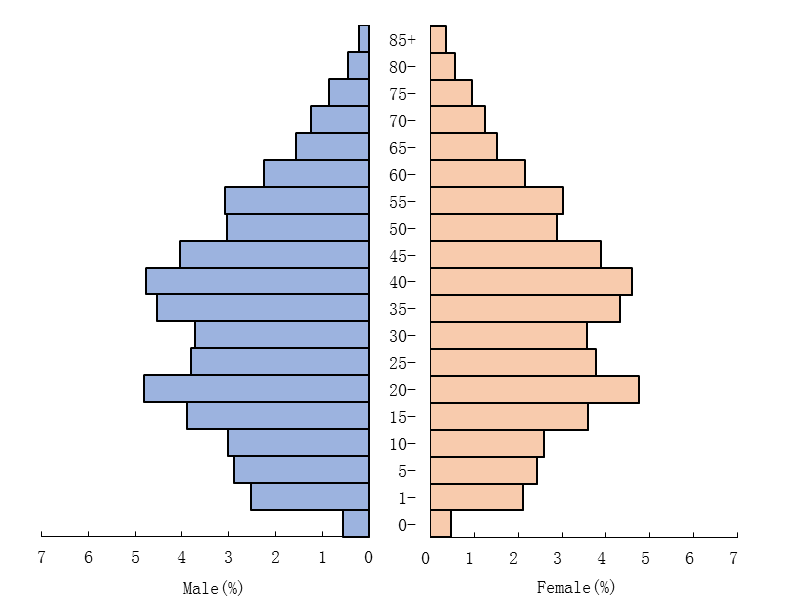
**

Supplementary Material 2

Ape-specific incidence and mortality rates by histology subtype of peripheral T-cell lymphoma in Beijing from 2007-2018 (1/100,000)

| Agegroup | Incidence | | | | | Mortality | | | | |
| --- | --- | --- | --- | --- | --- | --- | --- | --- | --- | --- |
|  | PTCL, NOS | AITL | NK/TCL | ALCL | Others | PTCL, NOS | AITL | NK/TCL | ALCL | Others |
| 0-4 | 0.06 | 0.00 | 0.00 | 0.05 | 0.00 | 0.02 | 0.00 | 0.00 | 0.03 | 0.00 |
| 5-9 | 0.02 | 0.00 | 0.00 | 0.08 | 0.06 | 0.02 | 0.00 | 0.02 | 0.00 | 0.02 |
| 10-14 | 0.04 | 0.00 | 0.04 | 0.13 | 0.00 | 0.00 | 0.04 | 0.02 | 0.04 | 0.02 |
| 15-19 | 0.08 | 0.00 | 0.06 | 0.00 | 0.05 | 0.05 | 0.00 | 0.00 | 0.02 | 0.06 |
| 20-24 | 0.09 | 0.01 | 0.02 | 0.04 | 0.03 | 0.07 | 0.00 | 0.00 | 0.01 | 0.01 |
| 25-29 | 0.05 | 0.01 | 0.06 | 0.02 | 0.05 | 0.02 | 0.01 | 0.00 | 0.01 | 0.02 |
| 30-34 | 0.07 | 0.00 | 0.05 | 0.04 | 0.02 | 0.05 | 0.00 | 0.02 | 0.01 | 0.02 |
| 35-39 | 0.08 | 0.06 | 0.04 | 0.09 | 0.04 | 0.05 | 0.01 | 0.03 | 0.04 | 0.01 |
| 40-44 | 0.12 | 0.03 | 0.10 | 0.02 | 0.06 | 0.04 | 0.00 | 0.04 | 0.01 | 0.03 |
| 45-49 | 0.15 | 0.07 | 0.11 | 0.04 | 0.04 | 0.14 | 0.04 | 0.04 | 0.01 | 0.03 |
| 50-54 | 0.15 | 0.15 | 0.12 | 0.05 | 0.06 | 0.10 | 0.06 | 0.04 | 0.01 | 0.02 |
| 55-59 | 0.25 | 0.13 | 0.17 | 0.03 | 0.11 | 0.15 | 0.06 | 0.08 | 0.02 | 0.07 |
| 60-64 | 0.32 | 0.25 | 0.22 | 0.06 | 0.14 | 0.25 | 0.16 | 0.10 | 0.03 | 0.05 |
| 65-69 | 0.47 | 0.29 | 0.12 | 0.12 | 0.16 | 0.38 | 0.22 | 0.06 | 0.03 | 0.07 |
| 70-74 | 0.67 | 0.39 | 0.05 | 0.09 | 0.14 | 0.48 | 0.23 | 0.00 | 0.07 | 0.09 |
| 75-79 | 0.76 | 0.39 | 0.20 | 0.12 | 0.20 | 0.53 | 0.35 | 0.10 | 0.06 | 0.12 |
| 80-84 | 0.68 | 0.43 | 0.21 | 0.06 | 0.31 | 0.61 | 0.28 | 0.12 | 0.06 | 0.18 |
| 85+ | 0.31 | 0.18 | 0.04 | 0.09 | 0.22 | 0.31 | 0.13 | 0.00 | 0.04 | 0.09 |

Supplementary Material 3

Histology type proportion changes of peripheral T-cell lymphoma in Beijing from 2007-2018(Cases, %)

| Year | PTCL-NOS | AITL | NK/TCL | ALCL | Others | Total |
| --- | --- | --- | --- | --- | --- | --- |
| 2007-2010 | 87(45.55) | 29(15.18) | 13(6.81) | 24(12.56) | 38(19.90) | 191(100.00) |
| 2011-2014 | 125(43.55) | 58(20.21) | 45(15.68) | 29(10.11) | 30(10.45) | 287(100.00) |
| 2015-2018 | 88(27.24) | 76(23.53) | 83(25.70) | 29(8.98) | 47(14.55) | 323(100.00) |
| Total | 300(37.45) | 163(20.35) | 141(17.60) | 82(10.24) | 115(14.36) | 801(100.00) |
